# From soil to seed: micronutrient movement into and within the plant

**DOI:** 10.3389/fpls.2014.00438

**Published:** 2014-09-05

**Authors:** Raul A. Sperotto, Felipe K. Ricachenevsky, Lorraine E. Williams, Marta W. Vasconcelos, Paloma K. Menguer

**Affiliations:** ^1^Programa de Pós-Graduação em Biotecnologia, Centro de Ciências Biológicas e da Saúde, Centro Universitário UNIVATESLajeado, Brazil; ^2^Departamento de Botânica e Centro de Biotecnologia, Universidade Federal do Rio Grande do SulPorto Alegre, Brazil; ^3^Centre for Biological Sciences, University of SouthamptonSouthampton, UK; ^4^Centro de Biotecnologia e Química Fina–Laboratório Associado, Escola Superior de Biotecnologia, Universidade Católica PortuguesaPorto, Portugal; ^5^Jonh Innes CentreNorwich, UK

**Keywords:** biofortification, mineral accumulation, partitioning, remobilization, transport, ubiquitination, uptake

The ability of roots to obtain micronutrients from the soil and to deliver these to the aerial tissues—including seeds—is essential to ensure that the shoot has the resources it needs to function effectively. However, plants need to control several steps during the journey from soil to seed, including uptake, transport, remobilization and storage. A better comprehension of the relative contribution of these processes, together with their overall coordination, is necessary for a more complete understanding of plant metal homeostasis and for the development of successful biofortification strategies. This Research Topic aims at addressing some of the most recent advances in micronutrient movement from soil to seed and to provide an overview of different approaches that can be used to generate micronutrient-efficient and biofortified plants. Here, we highlight some of the major points arising from these reports.

## Micronutrient uptake and transport

As the first step in the pathway of elements reaching shoots and seeds, micronutrient uptake from the soil is arguably the most studied aspect of metal and metalloid homeostasis. As such, identifying the mechanisms and proteins involved in transport into roots from the rhizosphere was key to further studies on metal movement within the plant. Iron (Fe) acquisition in plants, for example, has long been thought to be based on a reduction strategy (strategy I) in non-Poaceae but a chelation strategy (strategy II) in Poaceae species; however, recent data suggest that certain monocots, such as rice, (*Oryza sativa*) can combine characteristics of both. Ricachenevsky and Sperotto (2014) suggest two models of how these strategies were shaped through evolutionary time, and propose that the combined strategy might be common to more species than just rice. Jain et al. (2014) review the roles of Fe(III)-reductases, discussing Fe and copper (Cu) reduction at the rhizoplane, and the proposed roles for the reductases at leaf cell surfaces and in subcellular compartments.

When considering transport of micronutrients from the soil to the seed we need to understand the contribution of long distance transport, tissue and intercellular transport and the role of organelles. It is also important to consider the transport of toxic metals such as cadmium (Cd). The vacuole is a pivotal compartment in storing heavy metals (both essential and toxic), but also makes a substantial contribution to long-distance transport. Peng and Gong (2014) discuss the concept of vacuolar sequestration capacity (VSC), proposing it plays the role of a “buffering pool” not only controlling metal accumulation in cells but also dynamically mediating transport of metals over long distances. Certainly chelators such as phytochelatins and nicotianamine play an important role in regulating VSC. In rice, the membrane transporters VIT1 and VIT2 also appear to have a key role in long distance transport of zinc (Zn) and Fe between source (flag leaves) and sink organs (seeds) via the modulation of flag leaf Zn and Fe VSC. Other transporters implicated in the connection between VSC and long distance transport include AtMTP1, AtZIF1 (Zn) and AtCOPT5 (Cu). VSC is also key to the transport of toxic metals including Cd and arsenic (As). OsHMA3 sequesters Cd in root vacuoles and hence modulates shoot accumulation of Cd in rice. Heavy metal hyperaccumulator plants have reduced VSC in roots, promoting long distance metal transport and leading to metal accumulation in the shoots (Peng and Gong, 2014).

We are now starting to understand metal transport processes in a wider range of species. Palmer et al. (2014) provide a picture of the mineral transporter families and their expression over the life cycle in different tissues in switchgrass (*Panicum virgatum*), a C4 perennial grass that has great potential as a biofuel crop. Jung et al. (2014) report on the wild grass *Brachypodium distachyon* as a model for studying Cu transport in cereal crops, identifying CTR/COPT transporters and characterizing expressions patterns, subcellular localization and their function by growth complementation assay of yeast mutants under Cu deficiency. Some genes were transcriptionally up-regulated by Cu deficiency (*BdCOPT3* and *4*) and BdCOPT3, 4, and 5 conferred low affinity Cu transport. It was proposed that the increased sensitivity of some grass species to Cu deficiency could result from COPTs with lower uptake capacity and from an impaired partitioning among organs and tissues.

The uptake of other elements into roots and their transport within the plant are also discussed. Socha and Guerinot (2014) provide a comprehensive review of the transporters with potential roles in manganese (Mn) transport including information about their localization and regulation by Mn toxicity or deficiency. Molybdenum (Mo) metabolism is presented by Bittner (2014), including transporters and Mo cofactor (Moco)-dependent proteins, as well as the yet to be fully understood crosstalk between Mo and Fe metabolism. Finally, the role of metals in nodulation and symbiotic nitrogen fixation is presented by González-Guerrero et al. (2014), providing an overview of the role of different elements, metalloproteins and metal cofactors for nodule formation and function, and discussing contributions of distinct transporters for metal uptake, accumulation and spatial distribution within the nodules.

## Mineral remobilization

Remobilization of reserves to supply seeds with minerals has been emphasized in previous studies, but the contribution of stored minerals to total seed content is unclear, highlighting the need for a better understanding of metal remobilization to improve metal use efficiency in the context of biofortification. Sankaran and Grusak (2014) conducted a mineral partitioning study in pea to assess whole-plant growth and mineral content and the potential source-sink remobilization of different minerals. They conclude that net remobilization of some minerals from different tissues into seeds can occur, but continued uptake and translocation of minerals to source tissues during seed fill is as important, if not more important, than remobilization of previously stored minerals. Using ^65^Zn, Impa et al. (2013) show that Zn-efficient rice genotypes have a greater ability to translocate Zn from older to actively growing tissues than genotypes sensitive to Zn deficiency. Actually, as proposed by Sperotto (2013), under Zn sufficient condition, grain Zn accumulation in rice occurs mainly through continued root uptake during grain filling stage, whereas under Zn deficient condition both continued root uptake and remobilization of Zn from source tissues contribute equally to grain Zn loading. A similar pattern is found for Fe remobilization under Fe deficient or sufficient conditions. However, Stomph et al. (2014) used ^70^Zn applications at different times during rice development and suggested that the major barrier to enhanced Zn allocation toward grains is between stem and reproductive tissues, and that simply enhancing root to shoot transfer will not contribute proportionally to grain Zn enhancement. Pottier et al. (2014) reviewed micronutrient remobilization from leaves to seeds, and suggested that autophagy (a well-known mechanism involved in nitrogen remobilization to seeds during leaf senescence) is also involved in metal recycling and remobilization.

## Micronutrient storage

The ultimate goal of biofortification strategies is to develop grains with higher nutritional value and lower content of non-essential elements for human consumption or animal feed. Mineral homeostasis in plants is a tightly regulated process, depending on metal transporter activity and specificity, which directly affects mineral seed loading. Khan et al. (2014) highlight the importance of a safe nutritional enrichment of grains by means of precision breeding and transport engineering. This review reports the current understanding of the mechanisms involved in plant translocation and distribution of non-essential toxic elements like Cd and As by the same transporters that otherwise move nutrients such as Fe, Zn and Mn.

An important issue regarding micronutrient movement in plants and storage into seeds is the chemical form in which metals circulate in and between cells. There has been a number of advances in the identification of ligand and metal-ligand complexes in plant fluids (xylem and phloem sap, apoplastic fluid and embryo sac liquid) and it is now considered unlikely that metals are present in plant fluids in significant quantities as free ions. Instead it is much more likely that they occur in less reactive chemical forms and that may play a major role in controlling mineral seed loading and accumulation. Álvarez-Fernández et al. (2014) provide information on available methods for sampling plant fluids and the associated advantages and disadvantages with different techniques.

Grillet et al. (2014) review the mechanisms of Fe transport from the root to the seed, emphasizing the current knowledge on the chemical forms of Fe transported between symplastic and apoplastic compartments, including phloem loading. Also the authors show that the Fe bioavailability in seeds varies widely across species mainly depending on tissue localization and storage forms of Fe. Vasconcelos et al. (2014) approached the same subject analyzing soybean plants overexpressing the *AtFRO2* iron reductase gene and mineral accumulation in source and sink tissues to determine whether the reductase activity is a rate-limiting step for seed mineral acquisition. When exposed to high Fe supply the transgenic plants had an increase in leaf and pod wall Fe concentrations by as much as 500%. However, the seed Fe concentration only increased by 10% suggesting that factors other than plant reductase activity are limiting the translocation of Fe into the seed.

As Zn deficiency is prevalent in many parts of the world, especially where there is reliance on a plant-based diet, there is great interest in increasing the level and bioavailability of Zn in the grain of cereal crops. Olsen and Palmgren (2014) present an overview of the processes occurring in the transport of Zn from uptake at the plasma membrane of root cells to accumulation in the seed, reporting what we know in Arabidopsis to what we are starting to learn in cereals. The mechanisms involved in phloem unloading and post-phloem movement of Zn in the developing seed are discussed with respect to the apoplastic barriers found in the Arabidopsis seed.

## Applications and outlook

Knowing the fundamental processes governing mineral uptake, transport, remobilization and storage brings fundamental scientific knowledge that is important *per se*. However, an important goal in the understanding of these processes is to devise practical strategies to solve tangible problems (either via optimized agronomical practices, novel molecular and conventional breeding strategies, or genetic transformation utilizing the most suitable candidate genes). The biofortification of plant foods is one such area of research that benefits directly from a better understating of the molecular players and their physiological functions in nutrient homeostasis. In the current issue, Borrill et al. (2014) provide an integrated review on the available molecular tools for enrichment of wheat with Fe and Zn, and show us that we finally have the necessary molecular tools to accomplish this goal. They also remind us that when devising the best biofortification strategy, we will need to be mindful of future climate changes, such as a rise in atmospheric CO_2_ (which will negatively impact Fe and Zn contents in the seeds). Also, the authors emphasize the need for better communication between breeders and other plant scientists to enhance Fe and Zn in the grain in a sustainable way. Relevant to this is the consideration of toxic metals in these strategies to achieve a safe nutritional enrichment of seeds. Two main strategies could be important here: increasing the selectivity of transporters toward essential elements, and re-routing non-essential elements to non-edible parts of the plant (Khan et al., 2014).

Another benefit of an improved understanding of mineral uptake and transport is linked with abiotic stress issues. Plants can suffer from a multitude of mineral deficiencies, with boron (B) deficiency being observed in various agricultural soils, where it severely limits crop production. Here, Uraguchi et al. (2014) present a possible solution, increasing the growth of tomato plants under B deficiency by heterologously expressing Arabidopsis BOR1. This approach could be expanded to other crop species suffering from B deficiencies or at least considered for future breeding strategies.

This special issue presents novel mechanisms, some of which have not been fully recognized thus far, but have an obvious impact on plant mineral dynamics. One of these mechanisms is ubiquitination, a post-translational modification involved in protein turnover. Recently, several publications have revealed that ubiquitination also has roles in nutrient utilization. However, the extent to which plants rely on ubiquitination for regulating nutrient transport and compartmentalization is still in its infancy. In a perspective article, Yates and Sadanandom (2013) highlight the importance of the role ubiquitin plays in a plant's ability to uptake and process nutrients, including recent advances in understanding how ubiquitin supports nutrient homeostasis by affecting the trafficking of membrane-bound transporters. It is possible that we need to become more mindful of ubiquitination processes in future strategies when further improving biofortification or abiotic stress tolerance.

Altogether, the work presented here documents recent advances in the study of membrane transporters, chelators and regulatory proteins. Now, the challenge is to move forward and to integrate this information for an improved understanding of how the individual components and processes work together in the long pathway of micronutrients from the rhizosphere to the seed.

### Conflict of interest statement

The authors declare that the research was conducted in the absence of any commercial or financial relationships that could be construed as a potential conflict of interest.
